# The Application of Massage in the Treatment of Dental Pathological Conditions

**Published:** 1891-03

**Authors:** W. F. Rehfuss


ARTICLE II.

### THE APPLICATION OF MASSAGE IN THE TREATMENT OF DENTAL PATHOLOGICAL CONDITIONS.

BY W. F. REHFUSS, D. D. S.

*Continued from page* 455.

What constitutes the doseage of massage is to be determined by the force and frequency of the manipulations, and the length of time during which they are employed.

As the success of the treatment depends upon experience, it is logical to suppose that an expert manipulator, working diligently and with judgment will accomplish more than one who is inexperienced in the methods. The number of manipulations average about sixty per minute. I work from two to fifteen minutes, according to the object which I wish to accomplish. In ordinary pericementitis I would require about eight minutes. Formerly, I manipulated the gum only on the one side of the tooth next to the cheek or lip; now I use massage on the gum on both sides of the tooth, the labial or the buccal, and palatine or lingual. The efficacy of the treatment is thus increased.

The importance of extreme caution and gentleness is not to be overrated, for therein lies the success. Question the patient frequently and learn if the manipulating causes pain, and regulate the intensity accordingly. If the manipulator used undue strength upon such a sensitive tissue as the oral mucous membrane, he would injure rather than benefit the part.

I will now consider the physiological effects of massage: gentle stroking, though soothing, is, in a physiological sense, a mild irritant to the superficial vessels. It causes a narrowing of their calibre and a stronger and swifter current in them, because of its stimulating influence on their muscular coats and vaso-motor nerves. By *effleurage* we increase the circulation in the blood-vessels and lymphatics, by reason of the mild irritation produced, which attracts the circulation to the part. When we apply the friction we squeeze the pathologically changed parts, and by carrying the diseased tissue into the healthy substances, exposing them to firm stroking, the products of inflammation are absorbed. By *tapotement* or percussion we cause an influx of blood to the superficial vessels and remove the stasis in the current, invigorating the parts.

Massage exerts a simultaneous influence upon all the tissues within its reach,—the blood vessels, lymphatics, nerves, etc. It causes a more rapid absorption of the products of waste, stimulates and hastens the sluggish circulation. The mechanical effect of centripetal stroking is to push along the circulation, and the collapsing vessels cause a suction power that aids the returning current. When we use pressure in stroking, we crowd and squeeze the tissues together, impeding, in a measure, the outgoing or arterial circulation in the part thus treated, because of the contraction caused by this pressure of the tissues upon them. Naturally an accumulation of blood takes place, and on removing this pressure, the increased volume of blood owing to its sudden liberation rushes onward with greater force into the partially emptied continuance of the arteries.

In heat and cold we have two valuable remedial agents. Providing other and more convenient methods of application were available, more therapeutic benefit could be derived from their use than is now accorded to them. When we apply moderate heat to a tissue, it causes a flow of blood to the part and dilates the blood-vessels bringing the blood, as well as those returning it. If of a high temperature it acts like cold, causing a contraction of the vessels, counteracting vaso-motor paralysis. Therefore, the theory in the application of heat, to gain the desired therapeutic effect, is to apply it of a moderate temperature, say 90° Far., and gradually increase the temperature. This is but the same principle as massage, for the intermittent momentary compression of massage causes a mechanical contraction and dilatation of the blood vessels, arterial, capillary, and venous, at every application, from sixty times a minute upwards, which is certainly sixty times oftener than could be produced by the variations of the caloric in the same time. Moreover, the aid to the returning circulation, by being pushed along by massage is greater than that produced by heat, which has a tendency to enlarge the area of stasis, while on the other hand, the pressure of massage over the effusions and exudations disperses them and remove the stasis. When we apply cold to an inflamed tissue we can reduce the pain and swelling, but instead of increasing the circulation and hastening the retaining current of blood as by massage, the current is lessened and the effused products are not easily absorbed because of this contraction of the vessels. Again, cold applications to the oral mucous membrane are not without their dangers, as they may suspend nutritive action and convert inflammation into gangrene, or cause sloughing.

The dental pathological conditions for which massage is applied, and the different stages when it is beneficial, will now be considered. According to the requirements of each individual case, massage may be of primary or secondary importance, of no use at all, or even injurious. As to the range of its usefulness, experience and observation have shown me that it is beneficial in local disturbances of the circulation and nutrition, either in their incipient stages or after the acute symptoms have passed away; in acute and chronic forms of periostitis, gingivitis, alveolar abscesses and neuralgias. Local congestions and irritation can be relieved by massage. In diseases of the peridental membrane the treatment depends upon the nature of the inflammation. The objective point in its incipient stages is to abate the swelling, disseminate the products of inflammation, remove the stasis in the circulation and restore it to its normal condition. To be effective, the treatment must be both tonic and sedative in its effects, and must be capable of promoting the absorption of serous effusions, and causing the nutrition to be raised to a normal standard.

In these cases the massage at first should be gentle, as a counter-irritant effect is desired to bring the blood in the superficial vessels into more active circulation, so that when such remedies as tincture of aconite, tincture of iodine, chloroform, etc., are applied to the gum, they are more easily absorbed. It is my usual practice to manipulate the gum before applying to it any remedy, because the blood supply is increased, insuring the absorption of more medicine than ordinarily. When I use massage in the treatment of periostitis, I do not resort to lancing, unless considerable local congestion is present, when it is advisable to use massage first, which increases the blood-supply and also removes stasis, so that when the gum is lanced the consequent flow of blood is greater and continues longer than usual. This is an advantage, because the flow of blood from a cut by the lance is not always sufficient to relieve the congestion.

When the tendency of the inflammation is towards the formation of pus we can, by vigorous and repeated massage, hasten it and cause an earlier discharge. Massage softens the tissues, and by reason of the heat created, tends to direct the pointing of the abscesses. In acute alveolar abscesses massage is beneficial after the discharge of pus has ceased and the healing process commenced. The prognosis is more rapid than when massage is not employed. It stimulates and attracts blood to the part, causing the products of waste to be absorbed and disseminated, and induces the formation of new granulations.

In some chronic alveolar abscesses remedies seem useless. In several of these cases, by massage I have aroused the languid circulation and caused the absorption of the products of waste. In the class of cases under consideration we have a sinus present and a continued discharge of pus. It is a passive condition. The tissue outside the boundary of pus is torpid. If we allow the granulations to remain in this condition they may contract and consolidate in time, causing a closure of the sinus but clinical experience demonstrates that the tendency is to take on a retrograde action and involve the surrounding tissues and alveolar process. We must take advantage of this passive or languid stage and cause a formation of new granulations. Massage fulfills the requirements of these cases. Antiseptic injections should in addition be used and the prognosis will be more favorable than ordinarily.

Frequently we have complaints from patients that in teeth which have been abscessed, but that are apparently cured, the sinus having closed and the discharge of pus ceased, there is a sense of tenderness and soreness when the finger is pressed over the root of the tooth. I have noted that this trouble occurs particularly in bicuspid teeth. In a number of cases of this kind, I have by massage entirely removed such soreness.

The etiology of neuralgia is an important factor in its successful treatment by massage. In the incipient and milder forms it is particularly beneficial. Neuralgias, whose origin can be traced to local irritations, climate changes, irritations and inflammation of the superficial vessels, yield more readily to massage than any other local treatment. When used between the paroxysms of facial neuralgia, it lengthens the intervals between them and the attacks reoccur with less severity. In neuralgia in nerves not too deeply seated, I consider massage an excellent remedy; otherwise I depend on internal medication. In the earlier stages it acts as a prophylactic, relieving congestion by causing a free circulation in the surrounding tissues. In severe neuralgias of the fifth pair of cranial nerves, it is the custom to resort to the surgical method of exposing the nerve and stretching or dividing it. Stretching has become the most acceptable remedy; the object is to relieve the tension and pressure of the nerve. As these methods do not always assure relief, I believe massage should be thoroughly applied and tested before resorting to such severe operations. The action of massage is similar to nerve stretching. In the stretching operation the nerve is released from the neighboring tissues which compress it, which lessens its irritability and changes the structure and circulation. Massage makes mild stretchings in the same manner, and might succeed where violent ones would fail.

In diseases of the dental pulp, I do not advise the use of massage recognizing that we have remedies that are infallible. However, if periostitis is associated with the pulpitis the manipulations will be beneficial.

I also advise the use of massage to allay inflammation and pain incident to infantile or first dentition.

In conclusion, my employment of massage during the past eighteen months, in over two hundred and fifty cases of various kinds of dental diseases, and with more than average successful results, certainly justifies me in presenting and advocating its therapeutic merits to the dental profession.—*Dental Mirror.*

